# Effect of a stepped-care trauma rehabilitation programme for migrants in Germany: the ‘NAT counselling intervention’ study protocol for a naturalistic parallel group trial with a regression discontinuity design for quasi-randomization

**DOI:** 10.1186/s12913-026-14071-7

**Published:** 2026-03-02

**Authors:** Liliana Abreu, Jule Beck, Anke Hoeffler, Brigitte Rockstroh, Johanna Sill, Maggie Schauer, Elisabeth Kaiser, Katalin Dohrmann, Anke Koebach

**Affiliations:** 1 Department of Politics and Public Administration, University of Konstanz, Universitatstrasse 10, 78464 Konstanz, Germany; 2 Department of Psychology, University of Konstanz, 78457 Konstanz, Germany; 3 vivo international e.V., 78430 Konstanz, Germany

**Keywords:** Refugee mental health, Psychological distress, Stepped-care model, Screen-to-counsel, NET Narrative Exposure Therapy, NAT Narrative Trauma Work, Trauma rehabilitation, Forced migration, Peer expert by experience, Trial design

## Abstract

**Background:**

Asylum seekers who arrive in their host countries worldwide present with an alarming burden of trauma and distress. Related mental ill health is a matter of concern and hinders the mobilisation of resources necessary for successful social adaptation and integration into host communities, creating a vicious cycle. Although effective treatment is available, services are only accessible when conditions become chronic and/or life-threatening. To overcome the manifold barriers to care, we aim to implement a screen-to-counsel, stepped-care trauma rehabilitation programme for asylum seekers upon arrival in Germany. Individuals are invited to a personal interview at the end of which they are offered a specific intervention, depending on the severity of their symptoms and level of support needed: [s1] watchful waiting; [s2] Narrative Trauma Work (NAT), adapted from Narrative Exposure Therapy (NET; Schauer, Neuner, Elbert, 2025), delivered by peer migrant counsellors; or [s3] treatment by a licensed clinical psychological expert. This study is initiated to assess the effectiveness of the NAT intervention and the sustainability of the overall program within in the German health service setting.

**Methods:**

To assess the effect of NAT, we will apply a quasi-experimental parallel group study with a natural waiting list control group (regression discontinuity design). Follow-up assessments will be conducted continuously up to 12 months later, in person and online. The outcomes of the study will include symptoms of depression, anxiety and posttraumatic stress, using the Refugee Health Screener (RHS-15, primary outcome). The study will also assess social and economic integration, and resilience, as well as successful application of the NAT counselling intervention, amongst other measures. Cost-effectiveness and sustainability will be assessed for the overall programme. Sustainability will be measured on the basis of counsellor participation and satisfaction using a mixed methods approach. The study will be conducted at various selected sites in southwest Germany.

**Discussion:**

This study addresses a crucial gap in mental health care provision and integration support for refugees and strategic decisions for its implementation. Moreover, the programme builds on the work and evidence for trauma-focused treatment in refugee samples in low-income countries, but also from internally displaced persons and trauma-exposed individuals in post-conflict settings.

**Trial registration:**

ClinicalTrials.gov Identifier: DRKS00033410, January 29, 2024.

**Trial status:**

Protocol version: 1.0, Dec 13, 2024; Planned first recruitment: Apr 1^st^, 2024; Planned completion: unspecified.

**Supplementary Information:**

The online version contains supplementary material available at 10.1186/s12913-026-14071-7.

## Background and rationale

In the last years, forced displacement has affected an unprecedented number of people around the world. According to the United Nations High Commissioner for Refugees [1], a total of 108.4 million individuals were forced to leave their homes and resettle in 2022. Mid-2024 the organisation counted 122.6 million, an increase of > 8% per year, of which 54.3 million sought refuge in another country [1]. This increase is fuelled by intensifying waves of conflicts alongside poverty and the consequences of climate change [2]. Despite its relatively small size, Germany ranks fourth amongst the host countries with around 2.1 million refugees. Germany experienced a 28% increase in asylum applications from 2021 to 2022, and 35% increase from 2022 to 2023 [3], with the majority of refugees coming from Syria (31.2%), Turkey (19.1%) and Afghanistan (14.3%) [1]. A critical barrier to successfully manage and integrate these individuals is the high level of trauma exposure and the related mental consequences that manifest at individual levels and significantly impact community and societal dynamics.

### Trauma exposure and mental sequelae in refugee populations

For refugees, the months-long journey to Europe, depending on traffickers and the benevolence of strangers, is marked by an increased risk of victimisation as well as perpetration of violence. Typically, forced migration is preceded by periods of suffering and deprivation due to war, conflict, torture, childhood adversity, gender-based violence, and/or natural disasters. Numerous studies demonstrate the high trauma load in this population [4–9]. Moreover, arrival in the host country is associated with multiple burdens such as cultural (mis)understandings, loss of role, status and income, family, friends and more together with the ongoing insecurity during the asylum process. Given the insecurity and absence of formal protection, it is no surprise that violent offending is also reported [4, 5]. Such cumulative exposure to life threats and deprivation as well as the perpetration of violence have a profound and lasting impact on the mental health of individuals [10, 11]. Hereby, the pathological memory formation is one of the key drivers of symptom trajectories that lead to clinically relevant suffering [12].

**Fig. 1:**
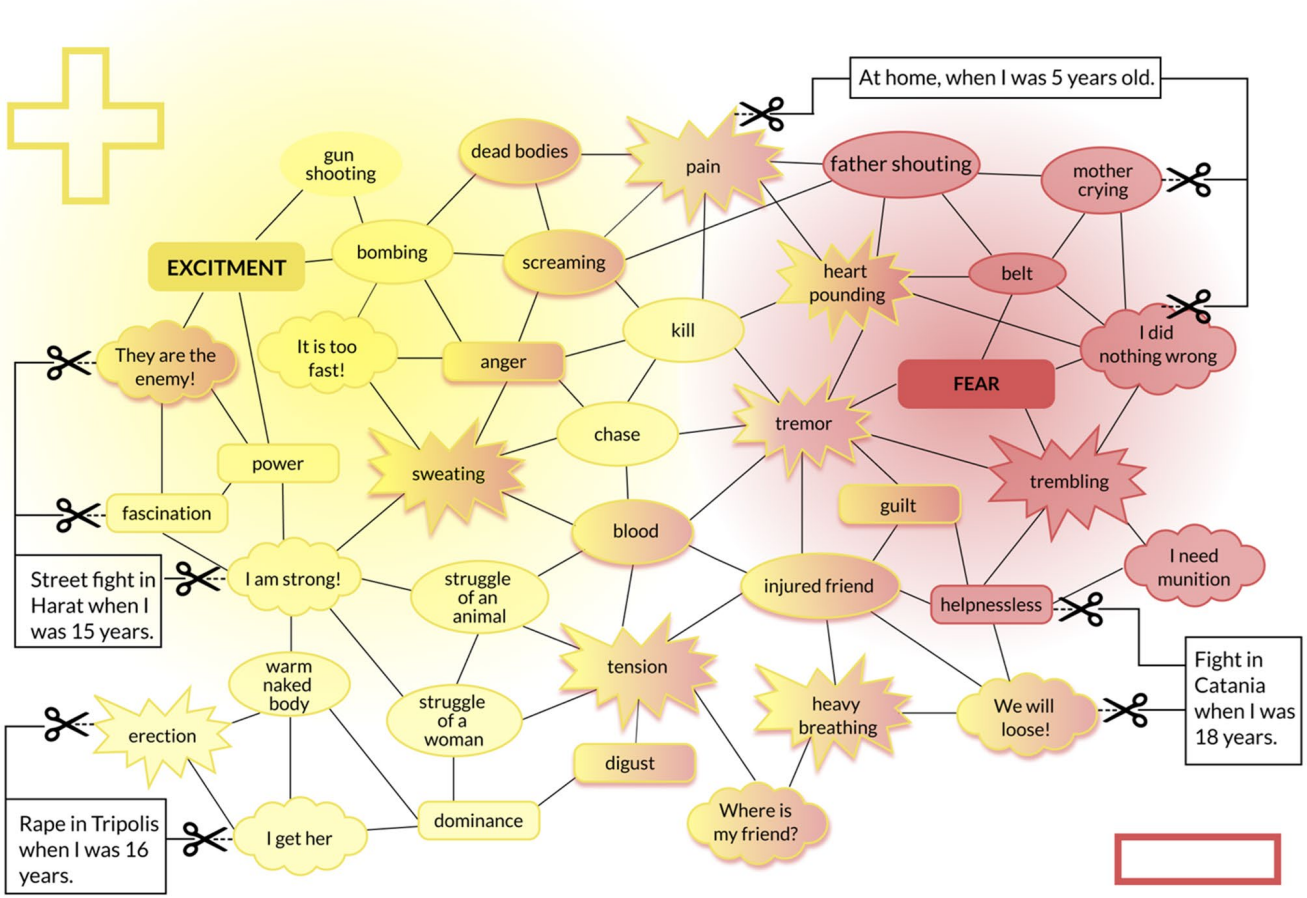
Hunting and fear memory networks (adapted from [18])

*Trauma memory.* During violent, potentially traumatic and life-threatening experiences, the process of storing and later retrieval of information is different from everyday life events: Emotional valence (negative/positive), interoceptive arousal, sensory input, and cognitions (implicit information) are stored in a way that they are very easy to recall but with deficient integration of spatiotemporal information [10, 13–15]. When encountering further threats, the individual’s reaction will follow familiar responses from the past and thus maximize survival chances [16]. However, as threats and triggers prevail, the memory may develop its own intrinsic dynamic and form a highly individualized, idiosyncratic neural network [17]. As a result of this, changes that are initially adaptive in a threatening situation, may result in psychopathological conditions, such as uncontrollable re-experiencing of implicit trauma memory components, compulsive avoidance or approach of reminders, changes in mood and cognition, hyperarousal, and aggression. When violent offending occurred alongside with victimization, the memories of those separate events are not stored together in a homogenous neural network of violence [13, 18, 19]. Namely, violent offending can be perceived as a fascinating and lustful experience [18]. Distinguished by the negative or positive valence experienced at the time of the event, two distinct associative memory networks are formed: a positive memory “hunting” network for perpetration and a negative “fear” network for victimization. Negatively experienced traumatic events and positively experienced perpetrated events are opposing but share salient commonalities (see Fig. 1; e.g., seeing blood or heart pounding). The psychophysiological responses that occur during both event types are similarly involuntary and innate [20]. Sensorial cues, like hearing screams or seeing blood, and interoceptive cues, such as rapid heart rate or respiration, are common in both victimization and perpetration experiences and interconnect the two networks. The higher the rate of exposure to such events, the more likely for their associative memories to be disconnected from their temporal and spatial contexts. Mnesic defragmentation occurs: the “what” is remembered, but the “when” and “where” are detached [10, 15, 21]. The aforementioned shared cues are distributed differently across the fear and hunting networks, depending on which network is more strongly reinforced and how. Specifically, negative reinforcement - where using sensorial or interoceptive cues in the hunting network of perpetrated events diminishes temporarily potential triggers for the fear network. For example, individuals with higher levels of appetitive aggression may be less likely to develop PTSD due to this effect (e.g. [22, 23]). The hunting network can also experience positive reinforcement due to the vinherent and intrinsic rewarding properties of violence [18, 24, 25].

Common psychological disorders among refugees and trauma-exposed populations include PTSD, depression, somatic symptoms, substance abuse, externalising/internalising symptoms in children and adolescents, and an increased propensity for aggressive behaviour [7, 11]. In Germany, it is estimated that approximately half of all newly arrived refugees meet the diagnostic criteria for at least one mental disorder [26]. Systematic reviews and meta-analyses estimated prevalence rates of ~30% for both PTSD and major depression diagnoses [27]. Increasing evidence further shows a heightened risk for involvement in crime (e.g. assault, street crime) in refugees [28]. In sum, the evidence suggests that refugees are at a particularly increased risk for developing the ‘classical’ trauma related sequelae such as PTSD, depression, delinquent/criminal behaviour and substance use disorders [27, 29, 30].

### Family relations and effects across generations

During periods of displacement (pre-flight, during flight, resettlement), the ability to meet the needs of all family members but especially those who need most protection – the children – is compromised. As this situation persists and periodically deteriorates, there is an elevated risk of substantial and pervasive ramifications of continuous and traumatic stress and mental ill health across generations [31, 32]. During recent years, studies have illuminated the trajectories from parental trauma and the related mental ill health to harsh parenting and its detrimental impact on offspring mental well-being (such as chronic depression and attachment disorders, anxiety, and others; e.g. [33–35]). For instance, in a case control study, Leys et al. [36] observed that Holocaust survivors with severe trauma-related symptoms exhibited family functioning patterns characterised by increased anxiety and reduced resilience compared to the control. The authors suggest that parental trauma has a harmful effect, impeding resilience and increasing intergenerational vulnerability to mental health disorders. Similarly, Peltonen et al. [37] highlighted the direct impact of maternal trauma on children’s emotional processing among a sample of 100 Syrian families (*N* = 212 children and *N* = 94 mothers) living in Turkey. Harsh disciplinary practices were associated with increased levels of anxiety and depressive symptoms in children. This highlights the far-reaching consequences of how parental trauma, compounded by displacement and resettlement stressors, impacts the mental health and the psychological integrity of the refugee offspring generation. In addition to these behavioural pathways of transmission, a related field of research suggests that violence experienced indirectly *in utero* by the mother, or directly during childhood or as adult, may lead to various severe and costly health and (epi)genetic (mal)adaptation in offspring [38–41]. In summary, the last decade has provided us with strong evidence to support the notion that violence breeds violence: organised violence spills over into the domestic, family and relationship spheres, setting in motion a powerful intergenerational cycle of violence that in turn undermines cohesion and trust [13, 42, 43].

### Social networks and marginalisation

The persistence of trauma related symptoms constitutes a significant impediment to the establishment and maintenance of relationships, while the concomitant marginalisation and discrimination exacerbate psychological symptoms. Taken together, the strength and density of social networks within host communities and the capacity of these communities to foster a supportive and inclusive environment has a significant impact on refugee’s mental well-being after resettlement [44, 45]. A number of cross-sectional studies with refugees have found an association between higher levels of psychological symptoms and lower levels of community participation. Namely, these studies have also highlighted related factors such as reduced social functioning, which includes communication difficulties and lack of social interest, and reduced social support [46–49]. A study of Sudanese refugees also found that those who perceived higher levels of social support had fewer symptoms of PTSD, anxiety and somatisation [50]. By providing a sense of belonging and mitigating feelings of isolation, social support can strengthen individuals' capacity to cope with the adverse effects of discrimination, thereby contributing to improved mental health outcomes [51]. In line with this, Ager and Strang [52] identify social connections as a core domain within their framework of refugee integration. The quality of these social connections can, for instance, significantly shape refugees’ perceptions and lived experiences of discrimination [53, 54]. However, while social support represents a critical protective factor, stand-alone support social support programmes are unlikely to sufficiently reduce traum-related symptoms [55–57].

### The economic costs of mental illness in refugees

Nightmares, intrusions and concentration difficulties, feelings of anxiety, anger, shame and other symptoms prevent refugees with poor mental health from functioning economically. In a prospective study, Dietrich et al. [58] found that mental health problems – especially when severe – affected the employment prospects for Syrian refugees living in Germany. Similarly, He et al. [59] conducted a cohort study in Sweden from 1960 to 1995 and demonstrated the increased vulnerability of the first generation of refugees to social and economic marginalisation. For instance, Straiton et al. [60] compared the sickness absence of migrants due to acute mental health problems with that of the native population over a 12-month period and showed that refugees had up to 7% more sick days than non-migrants. Consistent with this findings, Mastafa et al. [61] also found that mental illness increased broader societal costs (e.g. direct healthcare costs or indirect productivity costs). Therefore, the failure to provide effective intervention for refugees’ mental health will place a significant financial burden on tax-funded healthcare and social security systems.

### From past to current psychological treatment approaches for refugees

Common approaches applied for the treatment of trauma related disorders in refugees in high income countries have mainly included individual counselling or psychotherapy with ad hoc adaptation of modules from mainstream techniques such as cognitive behavioural or psychodynamic therapy to the psychological needs of the refugee client [62]. The enrolment rate of refugees into psychotherapeutic care is however very low [63]. Psychotherapists face various barriers to treating refugees, including linguistic, pragmatic, and structural considerations, as well as their own attitudes and competencies. Especially, refugees with predominant aggression rather than PTSD-related suffering would not be eligible due to an absence of diagnostic criteria. The authors also identified barriers to mental health care among refugees, namely, limited awareness for mental illness, fear of stigma, lack of knowledge of available services, and negative attitudes towards formal treatment [63]. For young male refugees with heightened levels of aggression, it is important to recognize that they may have learned to use aggressive traits as a coping mechanism for their trauma related suffering. This characteristic in and of itself may serve as a powerful deterrent to seeking counselling on the side of the refugee. Lending from Levesque’s process-oriented conceptual framework for access to healthcare [64], we recognize that strategic placement of psychotherapeutic services are to a large extent inadequate for refugees. Specifically, these services exhibit poor approachability, acceptability, availability, accommodation, affordability, and appropriateness. At the same time, refugees’ abilities to access care are also limited, including their ability to perceive (due to language barrier and cultural differences), seek (due to a new environment), reach (due to avoidance, scepticism, fear, practical issues), pay and engage (due to scepticism, norms, isolation). Recently, the inclusion of transcultural elements in psychotherapeutic approaches have gained prominence, but despite the increased availability of seminars on these topics (for e.g., in conferences, trainings, etc.) these efforts alone do not significantly improve the accessibility of psychotherapy services.

A special role within the mainstream psychotherapy approaches for refugees is played by Narrative Exposure Therapy (NET) [21, 65, 66]. About 25 years ago, in the context of organized violence, war and torture in Yugoslavia, Northern Uganda and Sri Lanka, a group of psychologists and researchers have developed NET [67]. The approach was grounded in culturally sensitive principles and universal neural mechanisms applicable to heterogeneous populations of refugees (and non-refugees), prioritising survivors' recovery needs and addressing multiple traumatisation [68–70]. With this approach, the developers broke with the then-prevailing taboo surrounding discussion of traumatic events, alongside prolonged exposure [71] and gave rise to a new era of trauma rehabilitation by giving victims a voice through testimony [68]. To allow for an efficient implementation in war-torn and resource-poor regions, NET is manualised, brief, and open access. Implementation by local counsellors without academic graduation in (clinical) psychology has become a pragmatic and successful *modus operandi* [72–77], including NET training-of-trainers schemes [77, 78]. Moreover, NET stands out for its ability to address severe and multiple traumatization across lifetime. While NET has been refined and adapted over the past decades, it has kept its outstanding procedural core structure and features, especially the theoretical framework which has been validated by numerous independent studies across various fields of psychotraumatology [14, 79–81]. Today, NET is considered one of the gold standard treatments of PTSD. The manual was translated in 12 languages and is used worldwide, universal applicability also allowed for the treatment if survivors in at risk populations, including survivors who are homeless or vulnerably housed [82, 83], who live in contexts of ongoing violence [84–87], or in psychiatric wards [88]. Clinical studies and randomised control trials (RCTs) with a broad population have confirmed significant treatment effects on posttraumatic stress symptom severity [89–93] as well as related problems such as depression [77, 94–96], borderline symptoms [94, 97, 98], somatization [99], physical health [70], pain symptoms [100], quality of life [101], guilt [72], shutdown dissociation and dissociative identity disorder [102, 103], aggression [86, 104], drug dependence [77], psychosis [105–107], disability [108], social dysfunction [86, 88, 104], chronic mental disorders [88, 109] and negative intergenerational effects [110]. Evaluations have further corroborated the benefits of NET by determining mechanisms of treatment effects on symptoms, such as improved top-down frontal cortical regulation of emotions and behaviour [111], improved immunological responses [99], and multiple epigenetic outcomes [112]. The benefits of NET not only appear shortly following completion, but also persist over time [90, 113]. The long-term effectiveness of NET deserves particular emphasis, and many (network) meta-analyses and reviews fail to capture this important aspect. NET was adapted for the special needs of children and adolescents [114, 115], perpetrators [84, 86, 116], communities [85, 117, 118] and online delivery [119, 120], and it is applicable and effective in a wide variety of samples without the need for further adaptation (see Box 1).

**Table:** Box 1

The mentioned studies have involved individuals of the following populations:
Refugees/migrants · torture survivors · low and middle income countries · post-conflict · gang members · ex-combatants · soldiers · survivors of gender based violence · individuals with diagnosis for borderline personality disorder · complex trauma · dissociative identity disorder · psychotic symptoms · other severe mental illness · pregnant woman · children with neurodevelopmental disabilities · elderly adults · individuals in insecure living conditions and ongoing violence · children and adolescents · gender diverse and intersectional groups / LGBTQ+ · individuals with intellectual disabilities · homeless persons or vulnerably housed · indigenous · survivors or trafficking

**Fig. 2:**
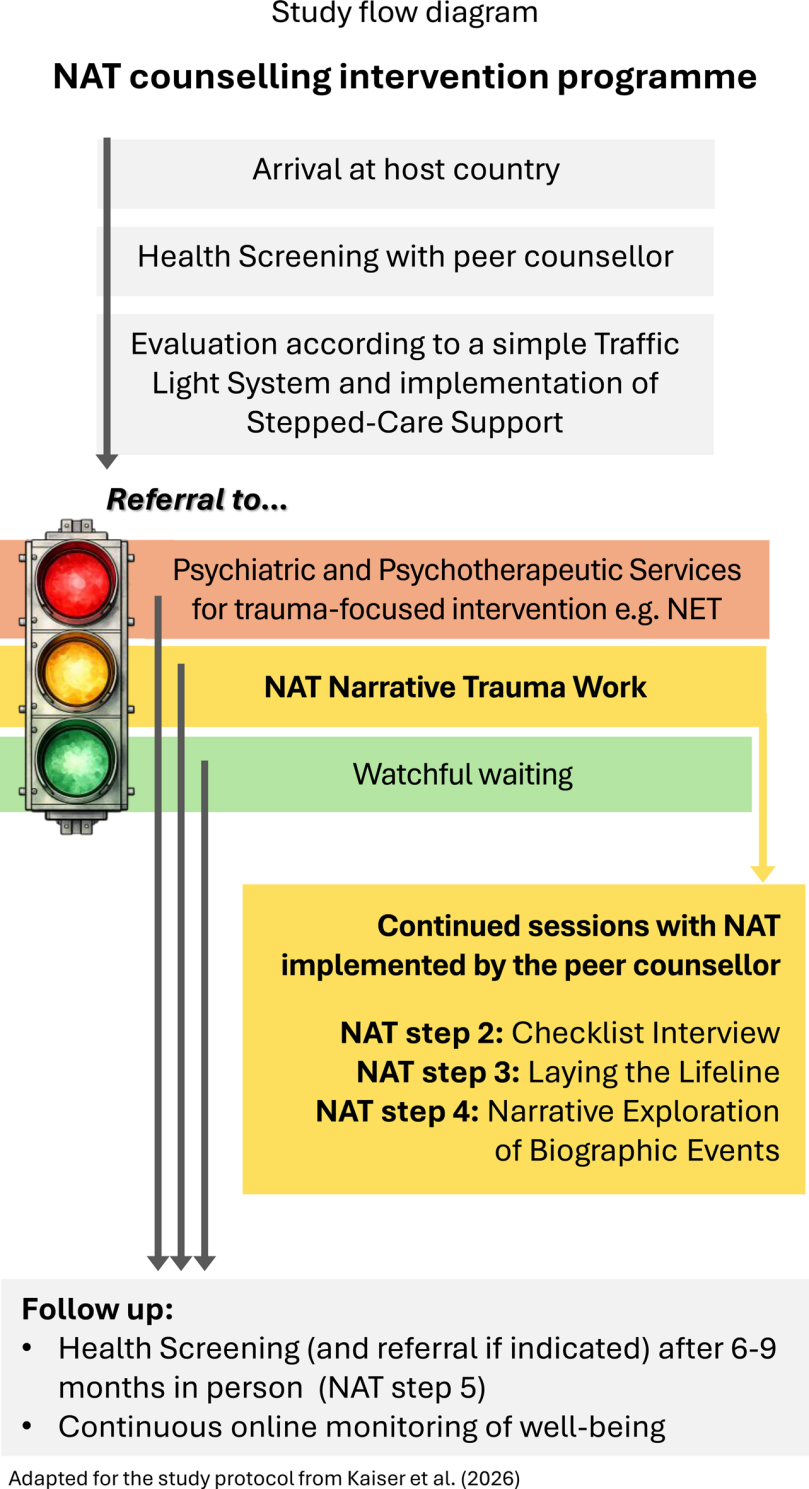
Summarized steps of the NAT counselling intervention programme

The overarching objective of this project is to pick up the key elements of this extensive knowledge gained from diverse countries of origin of refugees and integrate these into the German mental health system. To this end, we introduce a stepped-care trauma rehabilitation programme [121, 122] expanding the system with a peer counsellor component called *‘NAT counselling intervention’*, which integrates a screen-to-counsel approach for refugees residing in temporary accommodation in Germany (see Fig. 2). The specific goals of the ‘NAT counselling intervention’ are: (1) to invite asylum seekers to participate in a screening session conducted by a trained and assigned peer NAT counsellor with a similar cultural background, to assess potential psychological sequelae of traumatic events; the results of the screening are evaluated, and the client is categorized based on the logic of a traffic light system: red, yellow, or green and then referred to appropriate help, based on the severity of score identified by the 15-item Refugee Health Screener [123], a validated tool with excellent psychometric properties to screen for trauma related symptoms and probable diagnoses [124]; (2a) to deliver a specific counselling intervention called *‘NAT Narrative Trauma Work’* [125], adapted from NET and delivered by counsellors, for refugees with subclinical levels of symptoms (‘yellow’ group; RHS score of 13–36); and (2b) to facilitate the referral of refugees with a high likelihood of trauma related disorders (‘red’ group’; RHS score of ≥ 37) to a licensed psychotherapist^1^ for appropriate treatment, or (2c) to monitor and reevaluate cases with low symptoms; (3) follow-up; and (4) monitor psychological resources that enable individuals to participate in the community and society (‘well-being monitor’).

## Objectives

The objectives of this study are (1) to test the sustainability of the ‘NAT counselling intervention’ within the German health system and to identify characteristics that hinder or facilitate its scalability in terms of acceptability, adaptation, implementation and effectiveness [126], (2) to estimate the cost-effectiveness of the programme, and (3) to assess the effectiveness of the preventive and protective peer-lead intervention NAT Narrative Trauma Work [125] on participants’ mental health, integration and overall well-being.

## Trial design

The present study employs a naturalistic parallel groups design, integrating a regression discontinuity statistical approach for quasi-randomisation with a classical longitudinal design. The resulting pattern of evidence will indicate the sustainability, cost-effectiveness and effectiveness of the programme while we ensure that all study participants receive evidence-based treatment.

Assessments include an in-person baseline screening, monthly online or assisted self-assessments (hereafter: well-being monitor), and an in-person follow-up at 6–9 months post-baseline. Given the central role of the peer counsellors in the programme, we will assess their training and supervision in terms of acceptability, efficiency and motivations, through focus group discussions and survey-based evaluations. These evaluations will take place before the counsellors begin their active work and after they have completed a substantial number of NAT steps (screenings, referrals, checklist intakes, lifeline sessions and narrative event-telling sessions).

## Methods: participants, interventions and outcomes

### Study setting

The programme will be implemented in two medium-sized cities and surrounding areas of Baden-Württemberg, Germany, in collaboration with two local NGOs. The NAT counselling intervention will take place within the initial intake facility, where asylum seekers are waiting to be assigned to their long-term accommodation by the responsible district authorities. Invitation and initial information about the programme will be provided by social workers, visits and information sessions by the NAT counsellor in the accommodation, and flyers handed out to refugees.

### Eligibility criteria

*Refugees.* All adult asylum seekers (≥18 years) living in or arriving at the collaborating initial intake facilities are eligible to participate in the study and are offered NAT step 1, the screening interview with RHS-15 including feedback and referral if needed. Exclusion criteria include: severe cognitive impairment or communication problems that prevent participation in interviews, as well as individuals with severe intellectual or psychiatric disabilities who would qualify for guardianship (legal/ethical considerations).

For NAT steps 2 (checklist intake), 3 (lifeline laying) and 4 (narrative event-telling sessions), participants with an RHS score (items 1–13) between 13 and 36 points (‘yellow group’) are eligible; exclusion and referral to specialist care will occur in the case of acute psychiatric illness, acute suicidal ideation or danger to others, as well as acute and persistent intoxication that would prevent effective NAT sessions.

All steps of the NAT counselling intervention are carried out by trained counsellors under the supervision of NET experts (from vivo international e.V. and the University of Konstanz) or otherwise trained licensed psychotherapists. The NAT counsellors themselves are peers in terms of their cultural and migrant background and therefore have similar biographical experiences, moreover are familiar with and well-integrated into German culture and have good language skills (mother tongue and German) and strong social skills. It is recognised that conditions in the countries of origin vary and therefore the requirements for recruitment and inclusion of counsellors in the project programme have not been rigidly defined but are rather based on the real-world setting. However, high motivation, impartiality and strong integrative and social skills are prerequisites for participation in the NAT training programme. Exclusion criteria for counsellors include: suffering from a mental illness, holding extreme or radical religious or ideological views, have insufficient language skills or illiteracy in German. In addition, we provide a screening for PTSD symptoms in an initial session using the PTSD Checklist for DSM-5 [127, 128]. Following this assessment, participants are provided with an individualised information session on the potential psychological impact of the project and supported to make their own decision as to whether or not they are ready to train and work as a counsellor and to undergo counselling themselves. Due to the sensitive nature of this personal health information, no written records are made or kept.

### Who will take informed consent?

Prior to the initial screening, NAT counsellors will explain the programme and study details to potential participants. Written informed consent to participate will be obtained for both the screening and follow-up interview (see Appendix). Those eligible to participate in NAT will be asked to provide additional informed consent for participation in the intervention. All participants, regardless of their eligibility for NAT, will also be invited to complete the Well-being Monitor self-assessment. Separate informed consent will be obtained before the assessment begins. Finally, all refugees, irrespective of their level of distress and trauma symptoms, will be invited to complete the face-to-face follow-up.

### Additional consent provisions for collection and use of participant data and biological specimens

Not applicable as no biological data specimen will be collected.

## Interventions

### Explanation for the choice of comparators

In this naturalistic study, the following potential comparators to NAT (treatment condition) evolve: (1) NAT clients awaiting treatment; (2) clients with clinically relevant symptoms who are either (2a) waiting for psychotherapy or (2b) receiving psychotherapy; (3) clients without symptoms waiting for follow-up; and (4) clients before vs. after NAT (individual trajectories of symptom development, Table 1).

**Table 1:** Constellation of comparators

Treatment condition	Control condition	Limitation	Mitigation
[1] NAT	Awaiting NAT (RHS 13–36)	Reduced follow-up time points for those who wait for treatment; unknown N	-
[2] NAT	[2a] PT waiting [2b] PT receiving	Clients might still wait but may have started, or already finished PT; higher symptoms of the control group at baseline	Statistical control for the number of treatment sessions, use of the RDD
[3] NAT	Watchful waiting (RHS < 13)	Lower symptoms of the control group at baseline, low symptom levels generally	Evaluate number of clients who present with improving vs. deteriorating symptoms rather than
[4] After NAT	Before NAT	Within subject comparison; potential confounding factors	-

Note: RHS-15 = 15 item Refugee Health Screener with a sum score indicating symptom severity; PT = Psychotherapeutic sessions provided by a licensed Psychotherapist. Threshold to clinical relevance is suggested for ≥37. Watchful waiting is indicated for clients with ≤12 points

### Intervention description

The NAT counselling intervention programme begins with an initial screening using the 15-item version of the Refugee Health Screener [123, 124]; to assess trauma exposure and symptom severity in adult refugees. Based on their RHS scores, participants are allocated into three groups: low (RHS ≤ 12; green), moderate (RHS 13–36; yellow) and high (RHS ≥ 37; red) severity of trauma reaction; high levels indicate clinical relevance (Table 2).

**Table 2:** Allocation according to assessed trauma exposure and trauma reactions, taking into account safeguarding procedures

**No to low stress (RHS ≤12)**
Individuals in this category exhibit low trauma-, depression-, and anxiety reaction and good resilience. They are expected to adapt well and effectively cope with the challenges of daily life in Germany.
**Moderate stress (RHS 13–36 or no acute danger to self or others)**
Individuals in this category are unlikely to meet diagnostic criteria for trauma related disorders but are expected to have experienced trauma exposure. To protect these individuals from developing a trauma related disorder in the future, NAT counsellors will continue to provide step 2, 3 and 4 of NAT. In NAT, the counsellor guides the client in creating a chronological narrative of their life, focusing on traumatic experiences while also incorporating significant positive life events. This process allows clients to contextualise their network of sensory, cognitive, and affective memories of the past, thereby addressing the trauma memory structures. Moreover, NAT enables clients to reflect on their entire life journey, fostering a sense of personal identity and agency in the present ‘here and now’ and a positive projection of the client’s individual future. This approach conveys a notion of integration and a sense of achievement regarding their life in Germany. Implementing peer counsellors at this point leverages the ability to learn from role models while reducing cultural barriers such as language or varying interaction styles. Licensed psychotherapists and experts in psychotraumatology supervise the process, ensuring the correct application of the NAT intervention. In the event of worsening symptoms, a referral is being discussed based on a carefully developed protocol and set of criteria.
**Severe stress (RHS ≥ 37 or acute danger to self or others)**
Individuals in this category likely meet the diagnostic criteria for a mental health disorder or present an immediate danger to themselves or others. In such cases, individuals are referred to a licensed psychotherapist for clinical assessment and guideline-based treatment. Referrals are made to various service providers, including psychosocial centres, collaborating private practitioners, licenced psycho-therapists, outpatient clinics and hospitals. In cases of immediate danger to self or others, referrals for inpatient treatment may be necessary and directed to specialised clinical services in the region (psychiatric hospitals, acute care units, or, when appropriate, law enforcement).

### Criteria for discontinuing or modifying allocated interventions

In the event of danger to self or others, deterioration of trauma reactions or signs of other (comorbid) psychological symptoms, the client will be re-assigned to the red group and receive psychotherapeutic service or treatment at an acute care facility within the German health system. Thereafter, treatment delivery will follow the principles of the red group in full extent.

### Strategies to improve adherence to interventions

Counsellors will be provided with extensive supervision. Moreover, after conclusion of NAT, the counsellors will fill out a questionnaire asking for adherence to key principles of the NAT procedure for the specific client.

### Relevant concomitant care permitted or prohibited during the trial

All concomitant care that targets other than trauma related symptoms will be permitted.

### Provisions for post-trial care

Post-trial care will follow the regular public health system services.

### Outcomes

Outcomes of interest include the participants´ somatic and psychological symptoms, social integration, mental health literacy of the client, general life satisfaction, and post-migration stressors. Baseline and a follow-up assessment will be administered by NAT counsellors in face-to-face interviews and will assess psychological symptoms and social integration. Additional instruments are listed in Table 3 will be administered online.

**Table 3:** List of instruments of the well-being monitor

Outcome	Measure	Author	Admin.
Somatic symptoms	Somatic Symptom Scale-8 (SSS8)	Gierk et al. [129]	online
Psychological symptoms	Refugee Health Screener (RHS-15)	Hollifield et al. [130]	both
Life satisfaction	Satisfaction with Life Scale (SWLS)	Diener et al. [131]	online
Social networks	Lubben Social Network Scale (LSNS-6)	Lubben [132]	online
Social and economic integration	Immigration Policy Lab Integration Index (IPL-12)	Harder et al. [133]	both
Mental Health literacy Scale	Mental Health Literacy Scale (MHLS)	O’Connor et al. [134]	online
Resilience	Brief Resilience Scale (BRS)	Smith et al. [135]	online
Self-efficacy	General Self-Efficacy Short Scale-3 (GSE-3)	Doll et al. [136]	online
Food insecurity	Food Insecurity Experience Scale (FIES)	Cafiero et al. [137]	online
Food environment	Access to preferred food types	Adapted from Hadley et al. [138]	online
Aggression	Adapted version of the event scale of the appetitive aggression scale	Weierstall et al. [139]	both
Note: Both = to face-to-face interviews and online			

### Participant timeline


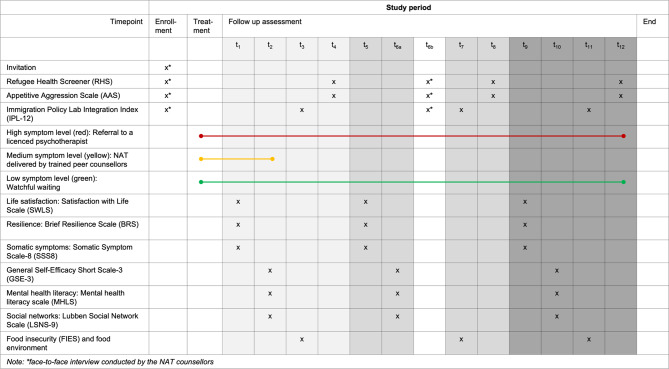


### Sample size

**Fig. 3:**
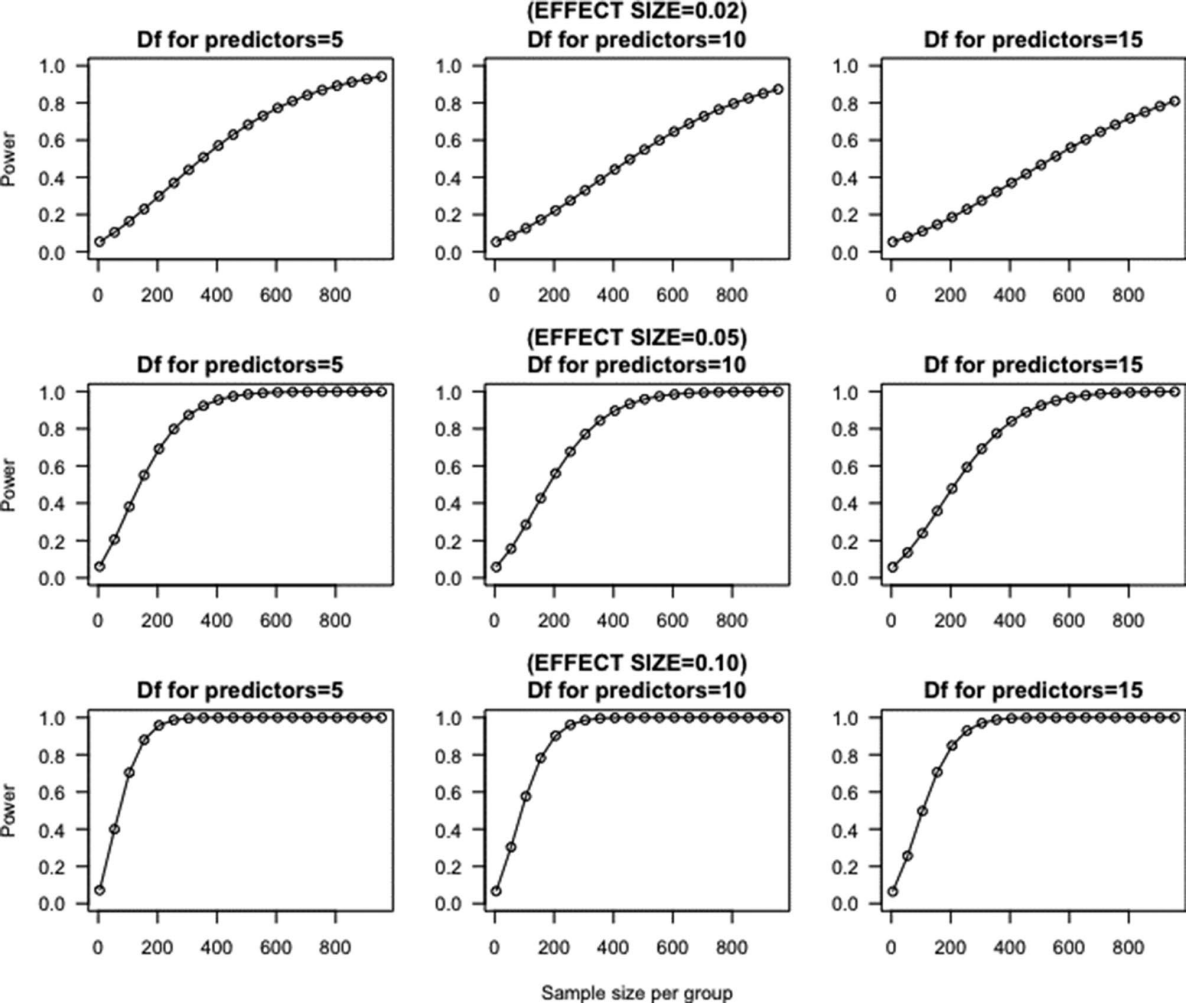
A priori power analysis estimation based on effect sizes of 0.02 (top), 0.05 (centre) and 0.10 (bottom) for a confidence interval of 95% and a sample size per group of 0 to 1000. Calculations were done using the pwr 1.3–0 package in R [140]

Approximately 1’500 refugees reside in the initial intake facilities at the collaborating sites and could potentially participate. We estimate that approximately 30–50% will participate in the study over the years. Figure A.1 shows the power of our study with variations in sample size, effect size and predictors. It is important to note that our sample size will follow the naturalistic parallel group design of our study and thus may differ from the estimated sample size (See Fig. 3).

### Recruitment

Social workers on site inform refugees in collaborating initial intake facilities and hand out flyers about the programme, including participation instructions. Additionally, NAT counsellors will be present in the facilities on a regular basis. With it, refugees who would like to participate can request to register for participation. Moreover, with bi-monthly visits at the initial intake facilities, public health experts and research assistants will invite refugees to participate in the online self-assessments of the Well-being Monitor.

## Assignment of interventions: allocation

Random allocation to treatment and control conditions is crucial in establishing causal inference. However, this approach is costly and raises ethical concerns when evidence-based treatment is existing and vulnerable populations are studied over extended periods. Therefore, the regression discontinuity design (RDD), introduced by Thistlethwaite and Campbell in the 1960s [141], serves as a powerful quasi-experimental alternative and has been applied in the field of counselling [142]. In brief, the RDD defines eligibility based on a *bandwidth* around a cutoff of a continuous variable that determines assignment. The premise underlying RDD is that participants near the cutoff are similar in all relevant respects except for treatment assignment, thus “as-if random” in this localized context. The *fuzzy* RDD also allows for deviation from the cutoff as criteria for single cases (systematic manipulation is however not allowed). With it, RDD provides an unbiased estimate of the local treatment effect, simulating a randomized controlled experiment for participants near the cutoff or within the bandwidth, respectively [141, 143–146].

### Sequence generation

Not applicable.

### Concealment mechanism

Not applicable.

### Implementation

**Fig. 4:**
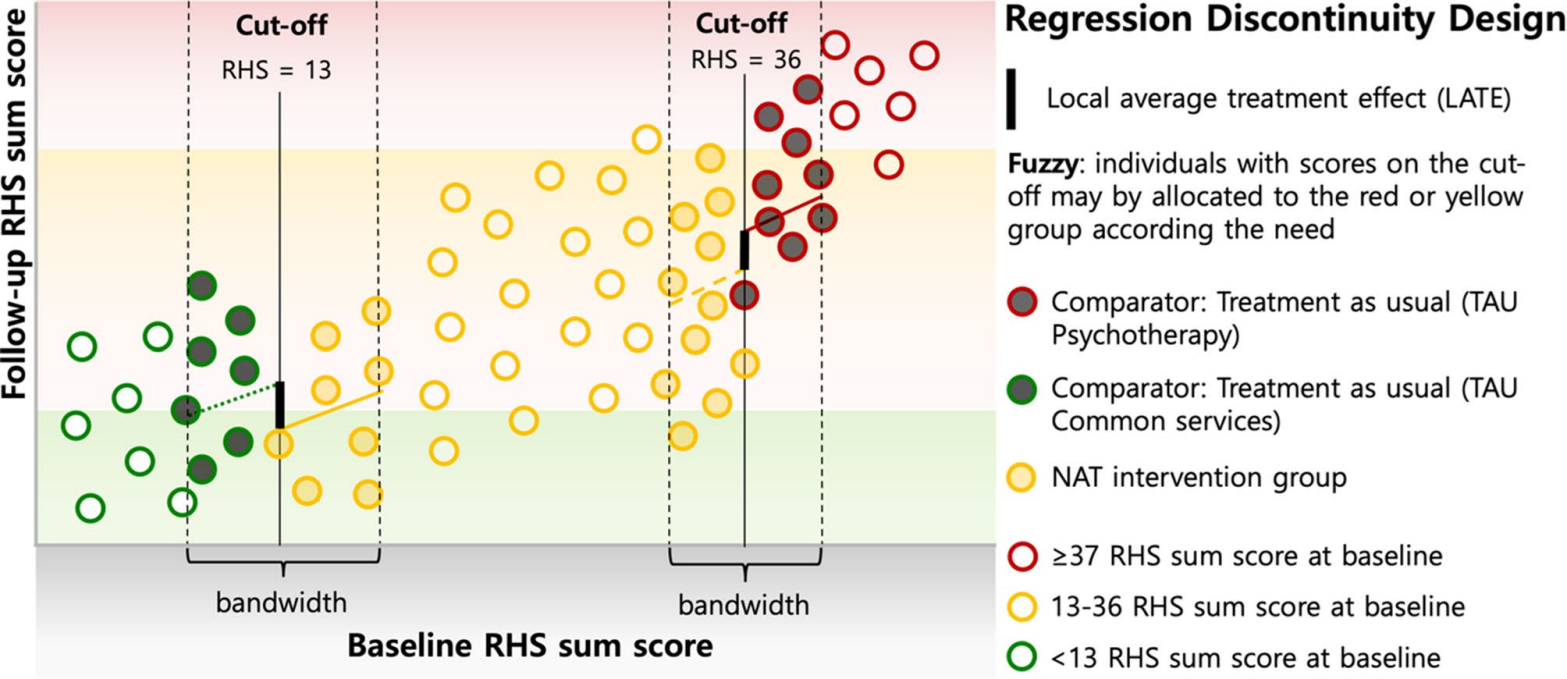
Hypothetical values of the NAT counselling intervention regression discontinuity design. RHS = Refugee Health Screen

To assign participants according to the RDD principles, we will use the RHS-15 (items 1–13) as eligibility criteria with two critical thresholds at 13 and 36 (see Fig. 4) as foreseen in the stepped-care model of the *NAT counselling intervention*. The RDD then assumes that the allocation to the conditions is nearly random within the determined bandwidth (RHS sum scores just above or below the cutoffs) and potential factors that would manipulate treatment effect vary randomly across participants in both groups. The *fuzzy* versus the *sharp* design version takes into account real-world implementation settings recognizing that participants with RHS scores in the yellow range need to be assigned to the red group, for instance, in cases of suicidal ideation or other negative psychological developments that requires treatment by clinical psychologist.

## Assignment of interventions: blinding

### Who will be blinded

Neither participants nor assessors will be actively blinded.

### Procedure for unblinding if needed

Not applicable.

## Data collection and management

### Plans for assessment and collection of outcomes

Data will be collected from the participating refugees and from the counsellors who deliver the treatment.

#### Assessment (refugees)

Data collection from refugees begins with a face-to-face interview conducted by one of the NAT counsellors (see Sect. Intervention description) and continues with 10-minute monthly online assessments (Well-being Monitor). Approximately 6 to 9 months after the initial screening, refugees will be invited to a face-to-face follow-up interview.

At the start of the face-to-face baseline interview, we will ask for demographic information, screen for trauma related symptoms, and collect information on social and economic integration. The follow-up interview after 6–9 months will re-assess levels of psychological distress and integration.

The 15-item Refugee Health Screener [123] will be used to assess the severity of trauma related reactions. The RHS was adapted from validated instruments for depression, anxiety and PTSD to screen for trauma related reactions in refugee populations. Each item is rated on a scale from 0 (not applicable at all) to 4 (extremely) and a total score of items 1–13 indicates the severity of symptoms (0–52) and the intensity of treatment required. Items ask for instance for nightmares, loss of interest or other trauma related symptoms. Items 14 and 15 are taken into account in a qualitative way. The RHS was recently validated with refugees in the German context [124]. Kaltenbach et al. [124] found high sensitivity and good feasibility, reliability and validity for both the self-rating and interview versions. Strong psychometric properties of the RHS were also found in other studies in Germany [147, 148] and elsewhere [149, 150].

An adapted version of the Appetitive Aggression Scale [139] for refugees will be used to assess aggression. The AAS is a 15 item semi structured interview that measures appetitive aggression according to the extent of agreement to statements, on a scale ranging from 0 (disagree) to 4 (agree) for each item. A total sum score will be used to measure severity of aggression (max 60).

Integration will be assessed using the short version of the Immigration Policy Lab Integration Index (IPL-12) [133]. The underlying concept is to identify and assess integration as both the knowledge and ability to thrive in the host society, rather than as mere assimilation. The index measures six dimensions of integration, namely psychological, economic, political, social, linguistic, and navigational integration. Various studies found a high construct validity and is broadly applicable to different refugee populations [151].

The 5-item Satisfaction with Life Scale (SWLS) [131]; is a widely used self-report questionnaire designed to measure an individuals’ subjective well-being and overall satisfaction with life. Participants are asked to rate each statement on a scale from 1 (strongly disagree) to 7 (strongly agree). The items of the SWLS items cover the following domains: *Standard of living*: The extent to which individuals feel that their standard of living meets their expectations and needs. *Life in general*: Overall assessment of life, including both positive and negative aspects. *Achievements in Life*: Perceptions of personal achievements, accomplishments, and success. *Relationships*: Satisfaction with social relationships, including family, friends, and community. The total score, obtained by summing the ratings for each item, provides a quantitative measure of an individual’s life satisfaction. Higher scores indicate greater life satisfaction, while lower scores indicate lower satisfaction. The SWLS is a concise and reliable tool for assessing subjective well-being and has been widely used in psychological research and clinical settings [131, 152–156].

The 8-item self-report Somatic Symptom Scale-8 (SSS8) [129]; will be used to assess the severity of somatic symptoms, asking whether the client suffered from fever, cough, diarrhoea, headache and other somatic symptoms in the last 14 days. A total score is calculated to indicate symptom burden, with scores ranging from 0 to 27, with higher scores indicating greater symptom burden. The high reliability and validity of the instrument has been demonstrated in large samples not only in Germany [157–159] but also elsewhere, including in self-administered online surveys [160–162].

Social integration will be assessed with an adapted 9-item version of the Lubben Social Network Scale (LSNS-9) [132, 163]; asking for kin and non-kin related ties as well as social networks with individuals born in Germany. The LSNS is particularly advantageous as it has been validated across various cultural contexts and has shown strong correlations with important health outcomes, including depression, mortality, and overall physical health, making it a robust tool for assessing social engagement among refugee populations [132, 163, 164].

Mental health literacy refers to the individuals’ ability to access and utilise mental health services effectively, and to recognize symptoms of mental health disorders. This facilitates prevention, early intervention, self-help, and implementation of evidence-based mental health care. We will use the Mental Health Literacy Scale (MHLS) [165]; to measure refugees’ knowledge and beliefs related mental health issues, focusing ontwo of its five domains: i) knowledge of mental health information sources and services (measured by items 16–19), assessing confidence in finding mental health information and accessing healthcare resources. And ii) beliefs and attitudes toward mental illness (measured byitems 20–28), assessing stigmatising attitudes and beliefs about mental illness, such as whether it’s a sign of weakness, if people with mental illness are dangerous, and willingness to seek professional help. Using this scale will allows us to assess refugees’ understanding of mental health issues and their attitudes toward seeking help, which can have a significant impact on the effectiveness of such intervention programme. This instrument has been applied in numerous studies with refugees demonstrating that individuals with higher levels of health literacy exhibit higher levels of health-seeking behaviours [166–168].

The Brief Resilience Scale (BRS) [135]; is a psychological instrument designed to measure an individual’s ability to withstand or recover from difficult circumstances in life. The scale consists of six items, and respondents rate their agreement with statements about resilience on a scale ranging from 1 (strongly disagree) to 5 (strongly agree). The BRS is a brief and widely used tool for assessing an individual’s capacity for resilience in the face of adversity. It focuses on aspects such as adaptability and the ability to maintain a positive outlook during difficult times [169–172].

The Food Insecurity Experience Scale (FIES) [138]; is a survey-based experiential measure. It is a globally used and validated measure of experiences and behaviours related to difficulties in accessing food [173]. Eight items ask about worrying about food scarcity, eating less nutritious food, eating fewer kinds of food, skipping meals, eating fewer types of food, running out of food, being hungry and going without food for a whole day. Each item refers to a 4-week recall period and can be answered with yes [1]/no [0]. The scale is validated using a scoring algorithm based on a Rasch model. Equating categorises respondents as food secure, mildly FI, moderately FI or severely FI [174]. Moreover, we will use different items to measure customization within the new food environment; indicating autonomy to practice preferred eating habits and thus, live one’s identity including cultural heritage. Previous studies have demonstrated higher levels of customization associated with integration and settlement [175, 176], as well as overall wellbeing through the increased capacity for self-agency and self-sufficiency [177].

#### Assessment (NAT counsellors)

Prior to the start of the intervention, the NAT counsellors will complete a demographic questionnaire, collecting information such as age, gender and years of formal education. To gain deeper insights into the counsellors’ motivations, experiences and expectations, we will conduct focus group discussions with all groups of counsellors. These qualitative data will provide a valuable context for understanding the counsellors’ perspectives and potential challenges they may face. Additionally, counsellors will rate their experience as active NAT counsellors by means of a standardized questionnaires (positive experiences and problems/difficulties and four questions about evaluation of having been adequately prepared, usefulness of supervision and their motivation to continue). After each NAT session, each NAT counsellor completes a one-page NAT-adherence protocol for each client.

### Plans to promote participant retention and complete follow-up

NAT counsellors will organise face-to-face follow-up sessions to ensure a personalised and supportive environment for participants. To increase engagement with the online assessment, public health experts and research assistants will be on site to support clients. In the event of a participant dropping out or deviating from the intervention, efforts will be made to collect relevant outcome data, allowing for a comprehensive analysis of the factors influencing participation outcomes, while respecting the participants’ right to withdraw from the study. In addition, participants will receive vouchers or cash as a monetary incentive (5€) for completing the online surveys, providing a motivational incentive for continued participation and completion of follow-up assessments. These retention strategies are designed to ensure data integrity and maximise the depth of insight gained from the study.

### Data management

All data will be collected with tablets using the software Qualtrics, after informed consent has been obtained from participants. We will adhere to data minimisation principles and collect only necessary information. Voice recorders will be used only for focus groups discussions with the counsellors. Once collected and upon connection with the internet, the data is uploaded to a secure cloud provided by Qualtrics. Qualtrics adheres to high security standards and all response data stored in the EU data centre will be encrypted using the industry standard AES-256/TLS protocol. Data transfer from Qualtrics to the University of Konstanz cloud (Nextcloud) will be secure. Downloaded datasets will be encrypted using Cryptomator prior to their storage. Access to the data will be strictly limited to authorised members of the research team, managed through role-based access controls. Data in the Qualtrics cloud will be deleted by the data manager (technical contact person) one year after the end of the data collection. The coding list will be deleted 10 years after implementation. Research data and back-up copies in the university cloud will be deleted after ten years, with master key files deleted one year after the end of the project. We will use pseudonymisation techniques to protect participants’ identities. Data quality will be ensured through regular checks, including checks for missing values, duplicates and plausibility of data ranges, according to a detailed data management protocol. We have conducted a privacy impact assessment in accordance with Art. 35 Datenschutz-Grundverordnung (DSGVO). We have established a data breach notification procedure to promptly address any potential data incidents.

### Confidentiality

All team members are informed and asked to sign a written commitment to high standards of confidentiality. This written commitment ensures that everyone involved in the study understands their responsibility to protect the privacy of participants. Clinical supervisors will be available to NAT counsellors to discuss client details, to ensure that sensitive information is handled appropriately, and that NAT counsellors have professional support when dealing with difficult cases. Senior implementation staff are responsible for regularly updating master key files and the coding list, ensuring that these files are encrypted and password protected. This centralised responsibility helps to ensure consistent and secure management of sensitive information. Physical records related to the trial are securely stored in a locked cabinet in a restricted access office. This additional layer of security for paper records complements the digital security measures described in the Data Management section. These strict confidentiality measures are designed to protect the privacy and rights of all participants and reinforce our commitment to ethical research practices beyond technical data protection measures.

### Plans for collection, laboratory evaluation and storage of biological specimens for genetic or molecular analysis in this trial/future use

Not applicable.

## Statistical methods

### Statistical methods for primary and secondary outcomes

#### Feasibility assessment

[1a-d] Descriptive statistics (frequencies, percentages, means, standard deviations) will be used to assess the feasibility of NAT and the NAT counselling intervention programme in general (Table 4). These quantitative measures will be complemented by qualitative data from focus groups conducted with NAT counsellors as participants. The focus groups will explore perspectives on the need for the programme, areas for improvement, perceived effectiveness, barriers and facilitators to implementation, participants’ experiences and suggestions for improvement (Table 4). Content thematic analysis will be used to analyse the qualitative data to provide a comprehensive understanding of the feasibility and potential for improvement of the programme, using Nvivo software for data analysis.

**Table 4:** Summarises the research questions and associated specific hypotheses

Research question	Alternative hypothesis	Null hypothesis	Ref.
Is the overall programme **sustainable**?	The majority ( > 50%) of refugees who leave the temporary accommodation have taken part in the project.	The minority ( < 50%) of refugees who leave the temporary accommodation have taken part in the project.	1a
	The programme is evaluated positively by stakeholders (refugees, counsellors, social workers).	The programme is evaluated negatively by stakeholders (refugees, counsellors, social workers) regarding issues that cannot be improved.	1b
	Recommendation to indicated treatment is followed by more than 50% of refugees.	Recommendation to indicated treatment is not followed by more than 50% of refugees.	1c
	The majority ( > 50%) of trained counsellor perform the screening and provide the intervention.	Only a minority ( < 50%) of trained counsellors perform the screening and provide the intervention.	1d
Is NAT **effective** (prevention)?	The number of participants with subclinical symptom levels at baseline who require specialised psychotherapeutic treatment after 6–9 months is lower in participants who received NAT than in the control group.	The number of participants with subclinical symptom levels at baseline who require specialised psychotherapeutic treatment after 6–9 months is equal in participants who receive the adapted NET compared to the control group.	2a
	The number of participants with subclinical symptom levels at baseline who do not require treatment after 6–9 months is higher in participants who received NAT than in the control group.	The number of participants with subclinical symptom levels at baseline who do not require treatment after 6–9 months is equal in participants who receive the adapted NET compared to the control group.	2b
Is NAT **effective** (symptom reduction)?	Symptom reduction in participants with subclinical symptom levels is stronger in participants receiving NAT than in the control group (see Table 1).	Symptom reduction in participants with subclinical symptom levels is the same in participants receiving NAT compared to the control group (see Table 1).	3
[4] Is the programme generally cost-**effective**?	The financial costs of the NAT counselling intervention are lower than those of other interventions.	The financial cost of the NAT counselling intervention is equal to or higher than other interventions.	4

[2a+b] Chi-squared tests will be used to compare the frequency of participants with subclinical symptom levels requiring more/less intensive treatment during the programme. Logistic regression analysis will be considered to estimate the odds of refugees with or without NAT requiring more/less intensive treatment (Table 4).

[3] To assess the effectiveness of NAT compared to the comparison groups (see Tables 1 and 4), we will combine the figures resulting from RDD with traditional approaches (GLM, clinical significance). In the RDD, we will calculate a local average treatment effect (LATE) of the intervention on primary (RHS-15) and secondary (LSNS, BRS, SWLS, SSS8) outcomes (see Table 1). To this end, we will firstly test whether the main criteria for a valid RDD are held. Using a graphical method (scatterplot), we will examine the relationship between the eligibility (RHS at baseline) and outcome (follow-up) variables. We expect the RHS score to be discontinuous at the cutoff values. In contrast, we do not expect to see discontinuities in relevant covariates at the cutoff, which would violate the assumptions of an RDD. Next, separate regression lines will be fit on both sides of a cutoff value using an ordinary least squares (OLS) regression as the starting point. Alternative functional forms will be tested with the goal of ensuring an unbiased estimation. The LATE will be computed directly as the difference between the regression lines at the cut-off (intercept). To test the statistical significance of the difference between the two conditions, we will use a *t*-statistic, dividing the treatment effect (difference between regression lines at cut-off) by the sum of the square of the standard errors of the comparison group (*SE*_*comparison*_) and the intervention group (*SE*_*interventio*n_). The calculated *t*-statistic will then be compared to a critical value from the *t*-distribution for the given degrees of freedom (*DF*). The degrees of freedom will be determined based on the number of the observations and the parameters estimated in the two separate regressions. In the most simple form, *DF = n*_*comparison*_ *+ n*_*intervention*_ *- 4*. The test will be two-sided. Moreover, we will run falsification tests and sensitivity analysis to ascertain the validity of the RDD [178], examine whether results are sensitive to the choice of bandwidth [144], run placebo tests on exogenous covariates and on the outcome at values other than the actual cutoffs [145]. Prespecified subgroup analyses will explore potential heterogeneity in treatment effects across different demographic and clinical characteristics. Adjusted analyses will account for potential confounding factors identified a priori.

Generalised linear mixed models (GLMM) with subgroups defined by the RDD will be used in addition and allow for integration of multiple follow-ups, if robust (*N*_min_ is sufficient to detect a small to moderate effect). Thus, to test the efficacy of NAT in comparison to our controls in regards to primary and secondary outcomes, we plan to use separate generalised linear mixed models (GLMMs) in R. To account for non-independence of measurements, we will include individuals as a random part of the models. To account for repeated measures, we will control the within-group variance for the effect of time, i.e. baseline and 3 and 6-month follow-ups, according to A Field, Z Field and J Miles [179]. To estimate the significance of the fixed terms, we plan to fit the models using maximum likelihood (ML), and compare models with and without each fixed term using likelihood-ratio tests [180]. In cases where the interaction term is significant, we will use post hoc Tukey tests using the R package emmeans [181] to assess which contrasts differ significantly. To correct for multiple comparisons controlling the false discovery rate, we will further adjust P-values accordingly [182].

Additionally, effect sizes will be estimated by calculating Cohen’s d [183] for within and between group differences following W Lenhard and A Lenhard [184]. Clinically significant change will be calculated using the reliable change index, following NS Jacobson, LJ Roberts, SB Berns and JB McGlinchey [185].

[4] We will estimate the cost-effectiveness of the NAT counselling intervention programme, respectively (Table 4). To this end, we will use cost-effectiveness analysis (CEA) based on the findings of the described trial and rely on established techniques from development and health economics [186–189]. In the first step, we will calculate the costs the NAT counselling intervention programme (as well as supervision, training, etc). Next, we will take into account prevented costs for specialised treatment, levels of improvement at symptom and daily life functionality (e.g. employment), and disability adjusted life years (DALYs) [190–193]. For each of the two compared strategies, a cost-effectiveness ratio (CER) will be calculated (cost versus outcome) allowing for an incremental cost-effectiveness ratio (ICER) which then allows for comparison of the NAT counselling intervention programme versus TAU and decision of superiority. We hypothesize that the NAT counselling intervention programme presents with a higher cost-effectiveness than TAU.

### Interim analyses

Our team will write interim reports at regular six-month intervals throughout the study. These reports will include a summary of project progress, key achievements, preliminary findings, challenges encountered, project timelines, resource utilisation, any necessary methodological adjustments and updates on participant recruitment, retention rates and data quality. The reports will be shared with the key stakeholders.

### Methods for additional analyses (e.g. subgroup analyses)

Not applicable.

### Methods in analysis to handle protocol non-adherence and any statistical methods to handle missing data

All eligible participants with reliable data and complete measures ( < 10% missing) at baseline will be included in the analysis. Participants who move into their long-term accommodation will remain in the study and follow-up will be continued. During the data collection periods we will give special attention to prevent missing data. One of the advantages of GLMMs over ANOVA is that it maximises the number of observations by excluding missing values only, rather than removing complete cases with some or all missing values present. This approach helps to maintain statistical power and reduce bias. In cases where missing data cannot be avoided, we use imputation methods using the mice R package [194] if more than 10% of the values in the entire dataset are missing, but no more than 30% per participant per assessment instrument. This threshold ensures that imputation is used judiciously and only when the amount of missing data is large enough to potentially affect the results, but not so large as to compromise the validity of the imputation process. We will conduct sensitivity analyses to compare results with and without imputation to assess the robustness of our findings. In addition, we will thoroughly document the patterns and reasons for missing data to inform the interpretation of results and discuss any potential biases introduced by missing data.

### Plans to give access to the full protocol, participant level-data and statistical code

The study protocol will be published in a peer reviewed journal. Participant-level outcome data and statistical codes may be shared in a public repository for replication under the condition that the anonymity of the participant can be guaranteed.

## Oversight and monitoring

### Composition of the coordinating centre and trial steering committee

The University of Konstanz (Germany) is responsible for the implementation of the study and scientific assessment following the protocol. The scientific part of the project coordination is carried out by two postdoctoral researchers (LA, JB) under the project leadership of BR, JS, AK and AH. Training for assessment and intervention as well as the supervision of the NAT counsellors will be led by BR, JS, EK. The project coordination team is further responsible for the supervision of the data collection and management as well as ensuring high quality of data management (JB). AH, TE, MS, EK will continue to provide advice, NET and NAT clinical consultancy and supervision on all aspects of the project.

### Composition of the data monitoring committee, its role and reporting structure

Due to the minimal risk of implemented interventions no data monitoring committee is established.

### Adverse event reporting and harms

The likelihood of severe adverse events (SAE) due to referral or narrative exposure is expected to be low, indeed this is the gold standard in international guidelines (e.g. NICE, APA and AWMF). In this study, SAEs are defined as adverse events that result in death, life-threatening injury or damage of self or others, persistent or significant disability or dysfunctionality, or congenital anomaly and/or require further interventions to prevent permanent impairment or damage. SAEs during NAT will be reported to clinical supervisors and case management discussed with the principal investigators. Any SAE attributable to NAT will be reported in the final publication.

### Frequency and plans for auditing trial conduct

Co-authors JB together with LA will audit the trial conduct, either in person or through trained representative moderators. The study status is reported to the project manager BR at least once a month. In addition, reports are shared with supervisors AK and AH, and regular meetings are held to discuss study progress.

### Plans for communicating important protocol amendments to relevant parties (e.g. trial participants, ethical committees)

Where appropriate, protocol amendments (e.g. changes to inclusion/exclusion criteria, outcomes, analyses) are communicated to relevant parties (e.g., Research Ethics Committee or Institutional Review Boards, trial participants, trial registries).

### Dissemination plans

Open access journals will be preferred for publication to facilitate the dissemination of results in low- and middle-income countries and the non-academic audience. We also plan to present the results in book chapters prepared for lay people and at congresses for the scientific community.

## Discussion

This study aims to evaluate the impact of the NAT counselling intervention programme, a screen-to-counsel stepped-care trauma rehabilitation programme designed to protect from the negative consequences of trauma and thus, improve refugee’s mental health and their sense of self-efficacy. The NAT counselling intervention introduces four novel elements into the refugee arrival and psychosocial care process: (1) Screen-to-counsel: The project invites refugees to participate in a validated trauma reaction screening as well as psychoeducation upon arrival in Germany to help them address their burdening ‘building block’ of adverse autobiographical experiences. (2) Stepped Care: Participants are offered appropriate psychosocial interventions according to their trauma exposure and reactions. (3) Peers and Intervention: A low-threshold, evidence-based, trauma memory-focused intervention (NAT counselling) is offered by migrant peer counsellors before psychopathology manifests, with the aim of identifying and treating trauma issues as quickly and effectively as possible and reawaken personal psychological resources, in order to enable the stabilisation of mental health, improve self-efficacy and strengthen migrants’ integration skills. This approach is thought to have the potential to effectively address the dual challenges of capacity and resource limitations in mental health provision for the growing population of refugees and forcibly displaced populations worldwide.

At the core of the NAT counselling intervention are (a) early identification of the trauma burden (screening) and (b) graduated help (depending of trauma burden), which includes NAT Narrative Trauma Work [125] or refugees with trauma related symptoms at subclinical levels of severity. NAT builds on the established principles of NET [21, 65, 66], a gold-standard intervention originally developed in the context of post-conflict and migration [90]. NET has demonstrated effectiveness in treating PTSD symptoms, depression, anxiety and other symptoms in refugees and other populations across the world with large effects long-term for PTSD [90, 93]. The use of non-health professionals has become a successful *modus operandi* in conflict-affected regions and LMIC, and ensures that the program is both resource-effective and accessible in contexts where access to qualified psychotherapists is limited [77, 78, 122, 124]. In NAT counselling, we use knowledge developed in the countries of origin of refugees and adapt this to the context of the German health system. This study will reveal whether the evidence holds for the high income, culturally western-based setting in Germany and provide crucial insights into its strategic set up. The NAT peer counsellor-facilitated model, with the combined screen-to-counsel and stepped-care approach are further novel elements to ensure an efficient mental health rehabilitation process when refugees arrive and thereafter. Here, we advance system-level features to improve approachability, acceptability, availability and accommodation, affordability and appropriateness but also address language barrier and other cultural differences. We ensure that the target population has the ability and support to seek treatment in the new environment (addressing issues such as avoidance, scepticism, fear, practical issues), ability to pay for, and get treatment, and engage with (scepticism, norms, isolation), to improve care.

With the naturalistic parallel group design with a waitlist control and a regression discontinuity statistical design for randomisation, we intend to comply with high scientific and ethical standards. This robust methodological framework enhances internal validity while maintaining feasibility in a real-world context. Comprehensive outcome measures – including somatic and psychological symptoms, social integration, mental health literacy, life satisfaction and resilience – provide a broad understanding of the intervention’s impact on participants. Additionally, focus group discussions offer valuable qualitative insights into participants’ perspectives on the acceptability and perceived value of the programme. The findings of this study have the potential to inform mental health policies and interventions aimed at improving the well-being and social integration of refugees. By demonstrating the sustainability, effectiveness, and cost-effectiveness of the NAT counselling intervention programme, the study contributes to the development of more equitable and accessible mental health care frameworks for vulnerable populations. Its innovative design also serves as a model for integrating trauma care into broader public health systems in high-income countries.

### Limitations

The innovative and naturalistic principles of the research design allow for a robust evaluation of the intervention, but also have certain limitations. First, the number of participants in each group cannot be accurately predicted, which may affect the robustness and generalisability of the results. Second, the degree of randomisation within the control groups is compromised, so comparative statistics need to be interpreted within the broader context and pattern of outcomes. This complexity is compounded by the comparison of an intervention (NAT Narrative Trauma Work) vs a programme (NAT counselling intervention). Third, the heterogeneous nature of the refugee population may introduce bias and/or limit the generalisability of the findings. Fourth, potential participants with highly stigmatising beliefs (e.g. young men from societies with strong patriarchal beliefs and norms) may be less inclined to engage in the programme. However, these challenges provide an opportunity to identify specific gaps and develop more targeted programmes and communication strategies to address the needs of these groups.

In conclusion, this study makes a significant contribution to the evolving field of refugee mental health by providing empirical evidence on the feasibility, effectiveness and cost-effectiveness of the NAT counselling intervention, stepped-care trauma rehabilitation programme. By addressing the critical need for accessible and effective mental health interventions for refugees, the study aims to improve both individual outcomes and broader social integration efforts.

## Electronic supplementary material

Below is the link to the electronic supplementary material.

Supplementary Material 1

## Data Availability

Parts of the final trial dataset and statistical codes will be available for replication on a public scientific repository or upon reasonable request, respectively.

## Footnotes

1 ‘Psychologische Psychotherapeuten’ in Germany and other German-speaking countries who has completed postgraduate training in cognitive-behavioural, systemic, psychodynamic or psychoanalytic therapy of at least three years’ duration, including theoretical and clinical components, and who is qualified to apply psychotherapeutic techniques to patients with mental disorders according to the ICD/DSM.

## Abbreviations

NET: Narrative Exposure Therapy
NAT: Narrative Trauma Work
RDD: Regression Discontinuity Design
LATE: Local average treatment effect
GLMMs: Generalised Linear Mixed Models
ANOVA: Analysis of Variance
DSGVO: Datenschutz-Grundverordnung
PTSD: Posttraumatic Stress Disorder
